# Biochemical markers for clinical monitoring of tissue perfusion

**DOI:** 10.1007/s11010-020-04019-8

**Published:** 2021-01-02

**Authors:** Marek Janotka, Petr Ostadal

**Affiliations:** Cardiovascular Center, Na Homolce Hospital, 15000 Prague, Czech Republic

**Keywords:** Microcirculation, Circulatory shock, Tissue perfusion, Hemodynamic monitoring, Oxygen saturation, Lactate

## Abstract

The assessment and monitoring of the tissue perfusion is extremely important in critical conditions involving circulatory shock. There is a wide range of established methods for the assessment of cardiac output as a surrogate of oxygen delivery to the peripheral tissues. However, the evaluation of whether particular oxygen delivery is sufficient to ensure cellular metabolic demands is more challenging. In recent years, specific biochemical parameters have been described to indicate the status between tissue oxygen demands and supply. In this review, the authors summarize the application of some of these biochemical markers, including mixed venous oxygen saturation (S_v_O_2_), lactate, central venous–arterial carbon dioxide difference (PCO_2_ gap), and PCO_2_ gap/central arterial-to-venous oxygen difference (C_a–v_O_2_) for hemodynamic assessment of tissue perfusion. The thorough monitoring of the adequacy of tissue perfusion and oxygen supply in critical conditions is essential for the selection of the most appropriate therapeutic strategy and it is associated with improved clinical outcomes.

## Introduction

**Fig. 1:**
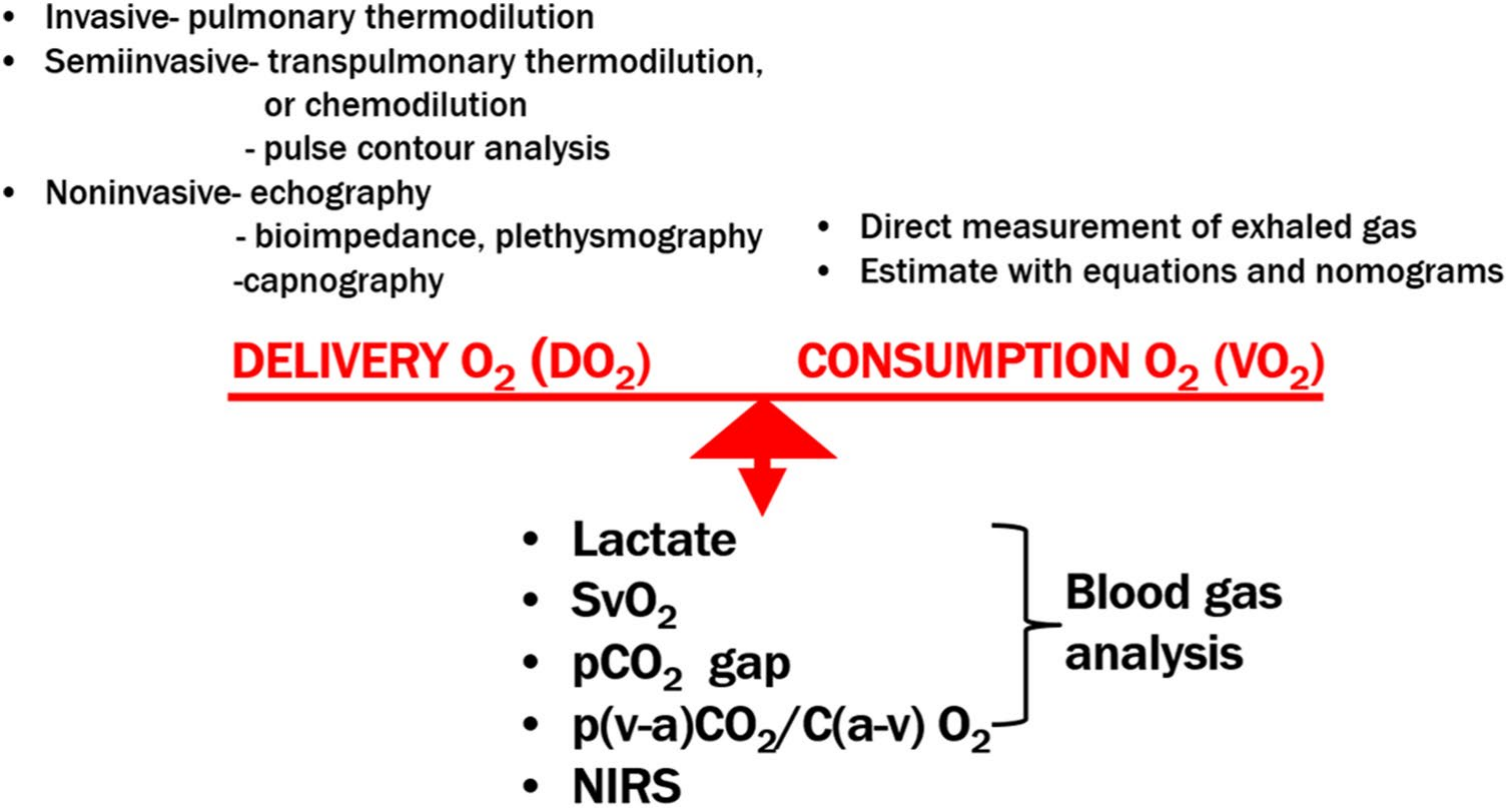
Complexity of the evaluation of global circulatory status. DO_2_, oxygen delivery; VO_2_, oxygen consumption; NIRS, near-infrared spectroscopy oximetry; PCO_2_ gap, central venous–arterial carbon dioxide difference; S_v_O_2_, mixed venous oxygen saturation

In individuals experiencing circulatory shock, it is essential to know whether cardiac output (CO) is sufficient to address tissue demands. Regardless of the type of shock, however, the ultimate consequences remain unchanged and have the same definition: a failure of oxygen (O_2_) utilization and cell metabolism caused by hypoperfusion resulting from circulatory failure—either the macrocirculation (heart and great vessels) or the microcirculation (capillaries, blood elements, cells) [1]. Hypoperfusion can be defined as a supply of O_2_ that does not adequately address the needs of cells [2, 3]. Failure of O_2_ use leads to anaerobic metabolism which is the source of several detectable products and byproducts. There is a broad spectrum of methods (from non-invasive to invasive) for measuring O_2_ supply for which CO is usually used as a surrogate in clinical practice [4] (Fig. 1).

However, it is difficult to measure O_2_ consumption because it can be estimated from nomograms or measured directly using exhaled gases; regardless, however, neither method is suitable for routine clinical use [5].

In treating any type of circulatory shock, sufficient CO must be ensured to fulfill tissue demands. CO is determined by heart rate (HR) and stroke volume (SV), according to the following equation:

$${\text{CO}} = {\text{HR}} \times {\text{SV}}$$

SV depends on preload, afterload, and contractility. In clinical practice, two approaches are used to increase SV (and CO): adding volume (to increase preload based on the Frank–Starling law) and administering agents with a positive inotropic effect (i.e., inotropes) to increase cardiac contractility. However, it is well known from clinical trials that the administration of higher doses of both volume and/or inotropes is associated with worse outcomes [6–9]. There is no rigorous threshold of CO that should be reached in treating circulatory shock, and the goal is to increase CO only as much as needed to ensure adequate perfusion [1]. The adequacy of perfusion (or hypoperfusion) is difficult to assess in clinical practice. Instrumental methods examining the microcirculation (e.g., videomicroscopic techniques) are not well established for clinical use [10]. Clinical signs of hypoperfusion are not very sensitive and manifest only in the later stages of shock [11]. Currently, the easiest way to assess the adequacy of perfusion and relationship between O_2_ supply and demand(s) is, therefore, the measurement of biochemical markers related to O_2_ metabolism (Fig. 1). However, the interpretation of the measured values requires understanding of the complex physiological principles in the context of other hemodynamic findings. The aim of our review was, therefore, to summarize current possibilities of the assessment and monitoring of tissue perfusion adequacy and interpretation of the values in different critical circulatory situations. The most frequently used parameters in clinical practice include mixed venous oxygen saturation (S_v_O_2_), lactate levels, partial pressure of carbon dioxide (PCO_2_) gap, and surrogates of the respiratory quotient (RQ). Evidence supporting the use of these parameters in individuals who experience septic shock is quite robust; however, they are also applicable to those who experience cardiogenic shock [2, 3]. Although sex and age may affect the course of shock (e.g., different immune response), it seems that these factors do not influence the clinical use of the parameters of tissue perfusion adequacy [12, 13].

### Global oxygen metabolism

Oxygen delivery (DO_2_) is expressed by the equation:

$${\text{DO}}_{{2}} = {\text{CO}} \times {\text{arterial}}\;{\text{O}}_{{2}} \;{\text{concentration}}$$

The major part of O_2_ in the blood is carried by hemoglobin (HGB). Only a clinically insignificant amount of O_2_ is physically dissolved and, therefore, is usually omitted:

$${\text{DO}}_{{2}} = {\text{CO}} \times \left( {{\text{concentration}}\;{\text{O}}_{{2}} \;{\text{bound}}\;{\text{to}}\;{\text{HGB}} + {\text{concentration}}\;{\text{O}}_{{2}} \;{\text{dissolved}}} \right),$$

$${\text{DO}}_{{2}} = {\text{CO}} \times \left( {{1}.{38} \times {\text{HGB}} \times {\text{S}}_{{\text{a}}} {\text{O}}_{{2}} + 0.00{31} \times {\text{P}}_{{\text{a}}} {\text{O}}_{{2}} } \right),$$

$${\text{DO}}_{{2}} = {\text{CO}} \times {1}.{38} \times {\text{HGB}} \times {\text{S}}_{{\text{a}}} {\text{O}}_{{2}} ,$$

where S_a_O_2_ is the saturation of HGB, P_a_O_2_ is the partial pressure of arterial oxygen, 1.38 represents the ml of oxygen bound to 1 g of HGB, and 0.0031 is the solubility coefficient of oxygen in plasma [14].

O_2_ consumption (VO_2_) can be calculated using the Fick principle (uptake of substance by an organ is proportional to the flow to the organ and arteriovenous concentration difference of the substance):

$${\text{VO}}_{{2}} = {\text{CO}} \times \left( {{\text{C}}_{{\text{a}}} {\text{O}}_{{2}} - {\text{C}}_{{\text{v}}} {\text{O}}_{{2}} } \right),$$

where C_a_O_2_ and C_v_O_2_ represent arterial and venous O_2_ concentrations, respectively, or

$${\text{VO}}_{{2}} = {\text{CO}} \times {1}.{38} \times {\text{HGB}} \times \left( {{\text{S}}_{{\text{a}}} {\text{O}}_{{2}} - {\text{S}}_{{\text{v}}} {\text{O}}_{{2}} } \right).$$

Under normal physiological conditions, O_2_ consumption depends only on the metabolic state (i.e., the higher metabolic rate the higher the O_2_ consumption) and is not influenced by DO_2_. This is based on the fact that DO_2_ greatly (up to five times) exceeds O_2_ consumption and serves as the delivery reserve for the body [15]. Therefore, under physiological conditions, VO_2_ is delivery independent and fluctuation in usual DO_2_ does not affect O_2_ consumption. There are two compensatory mechanisms for maintaining the equilibrium between VO_2_ and DO_2._ If O_2_ demands become higher, the compensatory increase in delivery will occur by increasing CO (first mechanism). If the increase in CO is not sufficient, then O_2_ extraction (EO_2_) from HGB (EO_2_ = S_a_O_2_ − S_v_O_2_) will rise (second mechanism). Increasing EO_2_ is associated with a decrease in S_v_O_2_. EO_2_ is approximately 25–30% in healthy resting conditions, and its possible increase provides a delivery reserve [16]:

$${\text{VO}}_{2} = {\text{HR}} \uparrow \times\, {\text{SV}} \uparrow \times\, 1.38 \times {\text{HGB}} \times \left( {{\text{S}}_{{\text{a}}} {\text{O}}_{2} - {\text{S}}_{{\text{v}}} {\text{O}}_{2} \downarrow } \right).$$

**Fig. 2:**
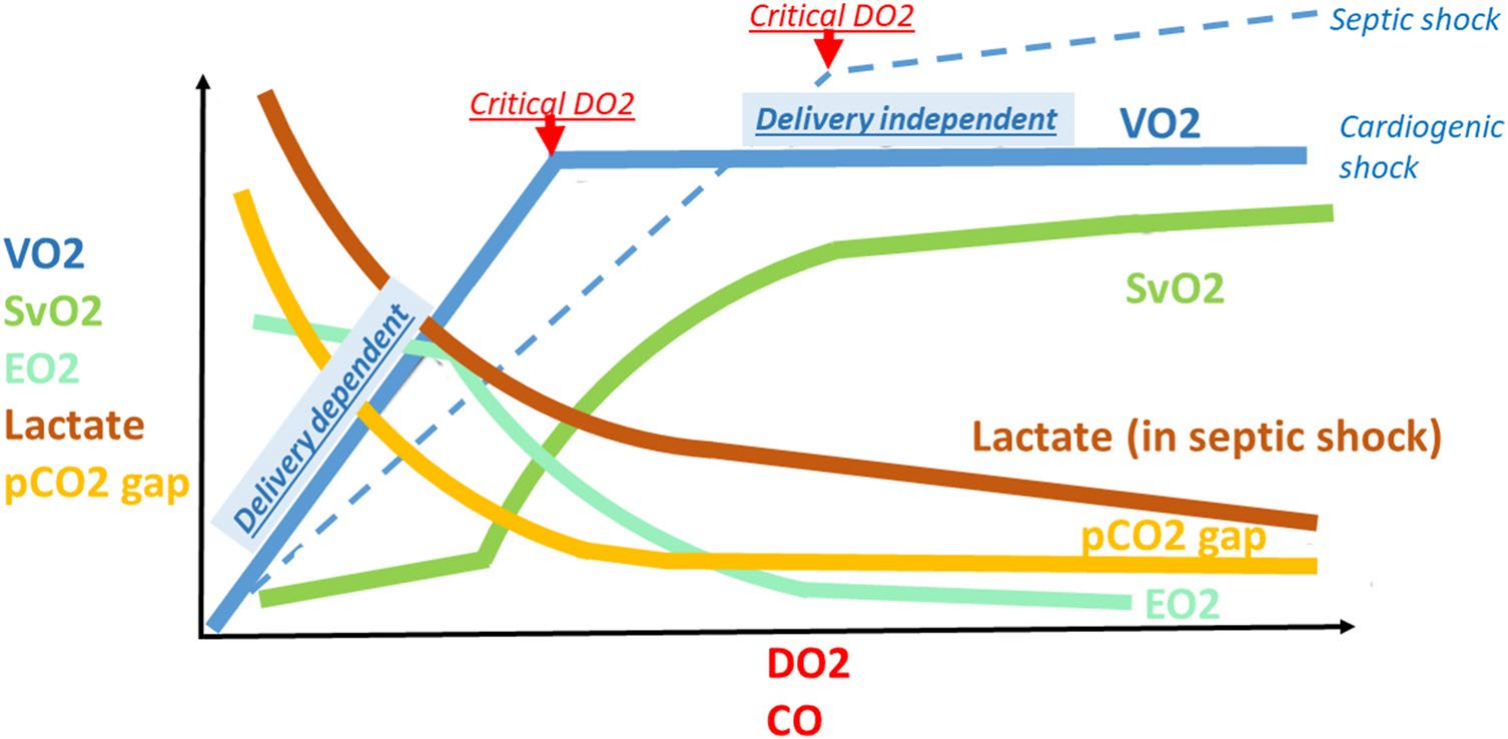
Relationship between levels of the parameters of global oxygen (O_2_) metabolism and O_2_ delivery (DO_2_) or cardiac output (CO). pCO_2_ gap, central venous–arterial carbon dioxide difference; S_v_O_2_, mixed venous oxygen saturation; VO_2_, oxygen consumption; EO_2_, oxygen extraction

If the capacity of an organism to increase CO is diminished or compromised (e.g., heart failure), DO_2_ can be further raised only by an increase in EO_2_. If this mechanism is also depleted (S_v_O_2_ decline to 50%), the critical DO_2_ (the least CO necessary to fulfill tissue demands) is reached and switched to adverse anaerobic metabolism with lactate production [14]. If both compensatory mechanisms are exhausted, O_2_ consumption becomes entirely dependent on DO_2_, and is known as delivery-dependent VO_2_ [14] (Fig. 2).

**Fig. 3:**
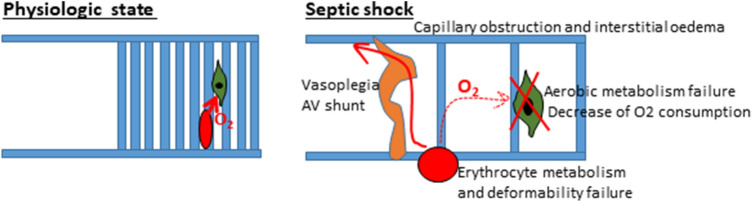
Difference between oxygen (O_2_) delivery at the microcirculation level in physiological conditions and in septic shock. Thrombosis and edema of capillaries and interstitial edema (due to increased permeability) lead to reduction in microcirculation net and prolonging of diffusion distance for O_2_. Ateriovenous shunts bypass oxygenated blood directly in the veins. Malfunction of oxidative enzymes lead to decrease in O_2_ use. Collectively, this induces the failure of O_2_ extraction

Primary failure of the macrocirculation (i.e., pump [heart] or great vessels [e.g., pulmonary embolism]) is known as cardiogenic shock and is a failure of DO_2_. Primary failure of the microcirculation and cell metabolism is known as distributive shock (e.g., septic shock) and is a failure of EO_2_ (Fig. 3). There is a higher level of critical DO_2_ in septic shock (dotted line in Fig. 2) due to failure of the microcirculation and cellular O_2_ use, which leads to malfunction of EO_2_ and a decrease in functional capillary density (heterogeneity of the capillary bed with good and poor perfusion). Therefore, some patients experiencing septic shock may benefit from increasing CO to higher values. However, this increase must be navigated by SvO_2_ and other parameters because routine increase to supranormal levels of CO may be associated with worse outcomes [7]. There can also be an uncoupling of the macro- and microcirculation when normalization of the macrocirculation (i.e., CO) does not improve microcirculation and cell metabolism [17–19].

## Venous blood oxygen saturation (S_v_O_2_, S_cv_O_2_)

Saturation of HGB by O_2_ in the venous blood can be measured in mixed venous blood (i.e., S_v_O_2_) in the pulmonary artery using a pulmonary artery catheter (PAC) or in central venous blood (S_cv_O_2_) using a central venous catheter (CVC) placed in the internal jugular vein or subclavian vein [20].

### Physiological principles

Generally, S_v_O_2_ values change with DO_2_ and EO_2_. SvO_2_ changes with DO_2_ (and CO) are non-linear (Fig. 2). There are only small changes in the independency zone from SvO_2_ > 70% (where compensation occurs through an increase in CO) and in dependency zone from SvO_2_ < 40% (where compensation through O_2_ extraction is depleted). The shape of the curve describing the relationship between S_v_O_2_ and DO_2_ is S-shaped and reflects the dissociation curve of oxy-HGB [20].

Normally, S_v_O_2_ values range from 65 to 80%. Generally, lower values imply low DO_2_ and a higher value means lower EO_2_ (Table 1). There are, however, more factors that lower S_v_O_2_ aside from low CO, including low HGB (anemia), low S_a_O_2_ (hypoxia), or high VO_2_ (i.e., O_2_ consumption). Increased consumption can be due to hyperthermia, shivering (thermogenesis), or cramps (epileptic seizure), increased consumption by breathing muscles while weaning from mechanical ventilation, or by psychomotoric agitation. If HGB concentration, arterial saturation, and consumption are optimized, then S_v_O_2_ depends solely on CO [21].

$${\text{S}}_{{\text{v}}} {\text{O}}_{2} \downarrow \, = {\text{S}}_{{\text{a}}} {\text{O}}_{2} \downarrow - \frac{{{\text{VO}}_{2} \uparrow }}{{{\text{CO}} \downarrow \times 1.38 \times {\text{HGB}} \downarrow }}$$

**Table 1:** Relationship between mixed venous oxygen saturation (S_v_O_2_) and adequacy of oxygen (O_2_) delivery

S_v_O_2_	Adequacy of O_2_ delivery
> 80%	Low O_2_ extraction, low O_2_ cell metabolism
65–80%	Normal O_2_ delivery → normal O_2_ extraction
50–65%	Low O_2_ delivery → compensatory increased O_2_ extraction
30–50%	Critical O_2_ delivery → O_2_ extraction depleted Switch to anaerobic metabolism
< 25%	Cell death

Higher S_v_O_2_ can be also caused by excessive DO_2_ or low consumption (e.g., sedation, myorelaxation, therapeutic hypothermia); however, it is rarely encountered and, therefore, high SvO_2_ is always an alert for EO_2_ or O_2_ metabolism failure. The possibility of cardiac disease with left-to-right shunt must also be excluded [14].

### Difference between S_cv_O_2_ and S_v_O_2_

**Fig. 4:**
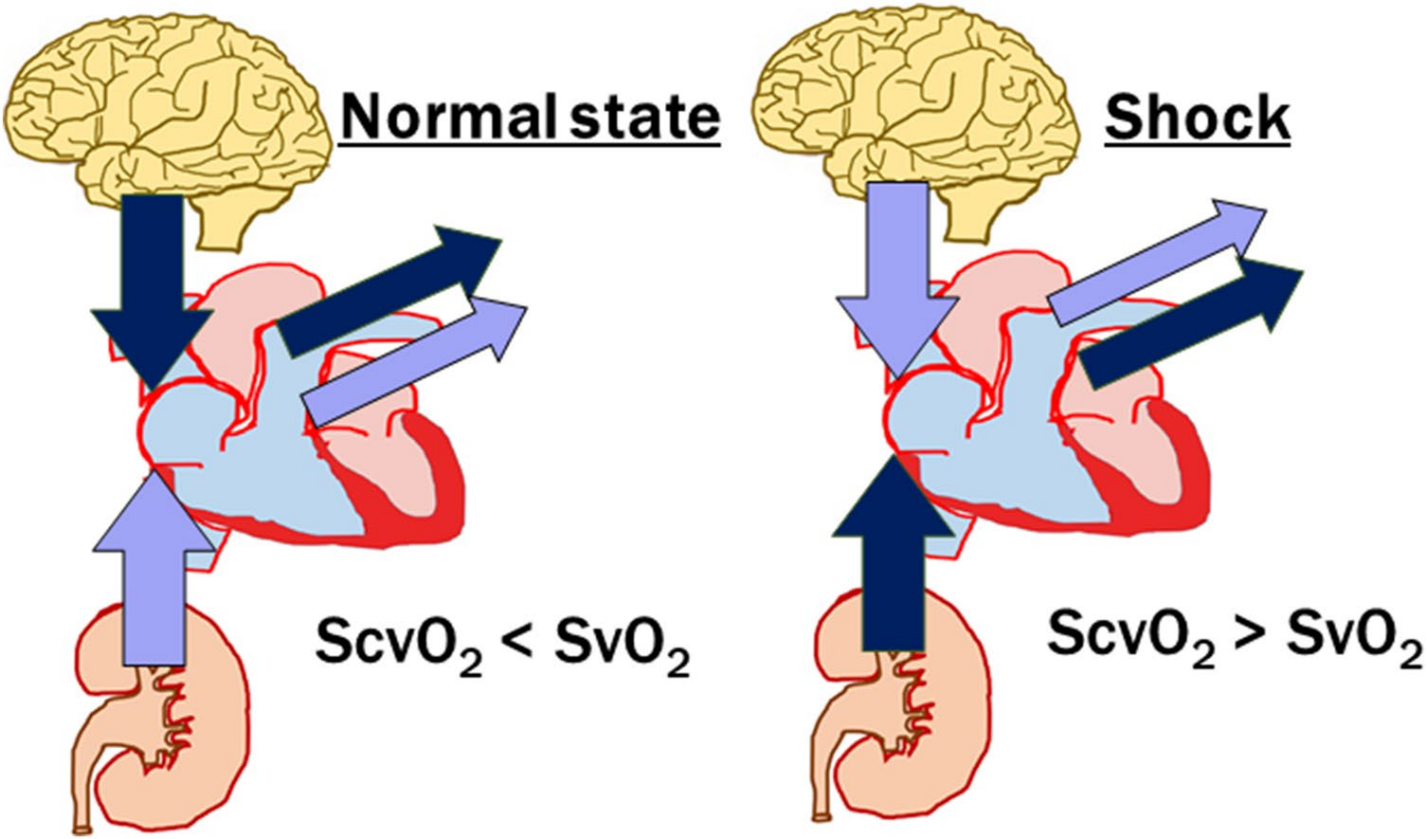
Explanation of the difference between saturation of hemoglobin by O_2_ in central venous blood (S_cv_O_2_) and mixed venous oxygen saturation (S_v_O_2_) under physiological conditions and in circulatory shock

SvO_2_ represents the saturation of HGB by oxygen in the mixed venous blood drawn from the pulmonary artery using a PAC, and contains blood from the superior vena cava, inferior vena cava, and coronary sinus. S_v_O_2_ is, therefore, a marker of global EO_2_ in the entire body. However, the insertion of a PAC is an especially invasive procedure with many potential risks. S_cv_O_2_ represents the saturation of HGB by O_2_ from the CVC, usually from the subclavian or jugular vein, and indicates regional EO_2_ from the upper part of the body under physiological conditions higher (due to high O_2_ demands of the brain) than in the lower part because of the inflow of highly oxygenated venous blood from the kidneys (renal blood flow is as high as one-quarter of CO). In healthy conditions, S_cv_O_2_ is generally 2–7% lower than in the mixed venous blood S_v_O_2_ containing blood from the kidneys (i.e., S_cv_O_2_ < S_v_O_2_). However, during circulatory shock, the situation is much different. Centralization of the circulation leads to vasoconstriction in the visceral organs, with decreasing perfusion and conserving blood for the brain. For this reason, EO_2_ in the lower part of the body is higher than in the upper part, and S_cv_O_2_ is higher than S_v_O_2_, which contains deoxygenated splanchnic blood (S_cv_O_2_ > S_v_O_2_). The difference increases with the severity of shock and can reach 18% [22, 23] (Fig. 4). There are more variables, such as the position of the CVC (S_cv_O_2_ and S_v_O_2_ become similar when a CVC is placed more distally in the right atrium) or lowering demands of the brain by sedation [24]. Although the absolute values of S_cv_O_2_ and S_v_O_2_ may differ, their trends are the same, and S_cv_O_2_ can be used as surrogate for S_v_O_2_ to assess perfusion adequacy [15].

### Measurement

S_v_O_2_ can be measured either intermittently from blood samples or continually using special catheters (either a PAC or CVC) equipped with optic sensors (light emitted from the tip of the catheter and reflected light from erythrocytes is measured using spectrophotometry). Although these systems need to be calibrated, they provide comparable values [25, 26].

### Clinical applications

#### Marker of tissue hypoxia (adequacy of CO)

The measurement of SvO_2_ is recommended by guidelines for CO adequacy monitoring [6, 27]. It provides information about hypoxia according to the amount of extracted O_2_. As mentioned above, the correlation between CO and S_v_O_2_ is worse in distributive shock (i.e., septic shock) due to extraction failure, and high S_v_O_2_ does not exclude low DO_2_. However, even in those states, if S_v_O_2_ is low, it means DO_2_ is low [1].

#### Prognostic markers and goal-directed therapy

Patients who experience septic shock have a worse prognosis when S_v_O_2_ is either low (< 65%) or high (> 80%) [6]. Until recently, S_v_O_2_ was recommended as a parameter for guiding the resuscitation of circulation in the early stage(s) of septic shock based on evidence of mortality reduction (guidelines from 2012 stated a goal of SvO_2_ > 65% and S_cv_O_2_ > 70%) [28]. The recommendations changed in 2016 after publication of three clinical trials that did not confirm the prognostic effect [29–31]. One reason is that baseline S_v_O_2_ may be high due to EO_2_ failure. However, there were other reasons for high baseline S_v_O_2_ in those trials than in previous trials; more specifically, patients were less sick and S_v_O_2_ was measured after initial volume treatment. Therefore, although the current recommendation for the use of S_v_O_2_ for goal-directed therapy (GDT) is not as strong as before, it is still suggested in patients with low S_v_O_2_ [1, 6].

#### Marker of incoming distributive shock (uncoupling)

A sudden unexplained elevation in S_v_O_2_ may imply the development of extraction (i.e., EO_2_) failure and microcirculation damage (e.g., systemic inflammatory response or septic shock) [14, 32].

#### Marker of catheter wedging

When using a PAC equipped with an optic sensor (described above), high S_v_O_2_ indicates wedging, either unintentionally when the catheter is placed too distally, or appropriate wedging during the measurement of pulmonary capillary wedge pressure [5].

#### Not a marker of local hypoxia

S_v_O_2_ is not a sensitive marker of local hypoxia (e.g., acute limb ischemia). In these situations, S_v_O_2_ will be normal due to the majority of blood with normal S_v_O_2_ originating from other organs [6].

### Near-infrared spectroscopy oximetry

Near-infrared spectroscopy (NIRS) oximetry is a non-invasive method that uses self-adhesive patches equipped with sensors (light emitter and sensors of reflected light are spaced several centimeters from one another) placed on the skin. The light penetrates several centimeters into the tissue and, using several algorithms, provides information regarding the status of oxygenation of HGB in the microcirculation (i.e., mixture of arterioles, capillaries, and venules (peripheral saturation, rSO_2_) 3–4 cm under the skin. Because the majority of blood is pooled in the veins, the value is driven, in large part, by venous saturation. Therefore, NIRS oximetry behaves in a manner similar to S_v_O_2_ (i.e., reflects CO) and is falsely high in conditions with O_2_ extraction failure (e.g., sepsis). Hypoperfusion is obvious when rSO_2_ < 50% or if there is a drop > 20% from baseline. It has been shown that NIRS oximetry values correlate with CO in cardiogenic shock. Currently, this method is increasingly used for non-invasive hemodynamic monitoring [33–36].

## Lactate

### Physiological principles

**Fig. 5:**
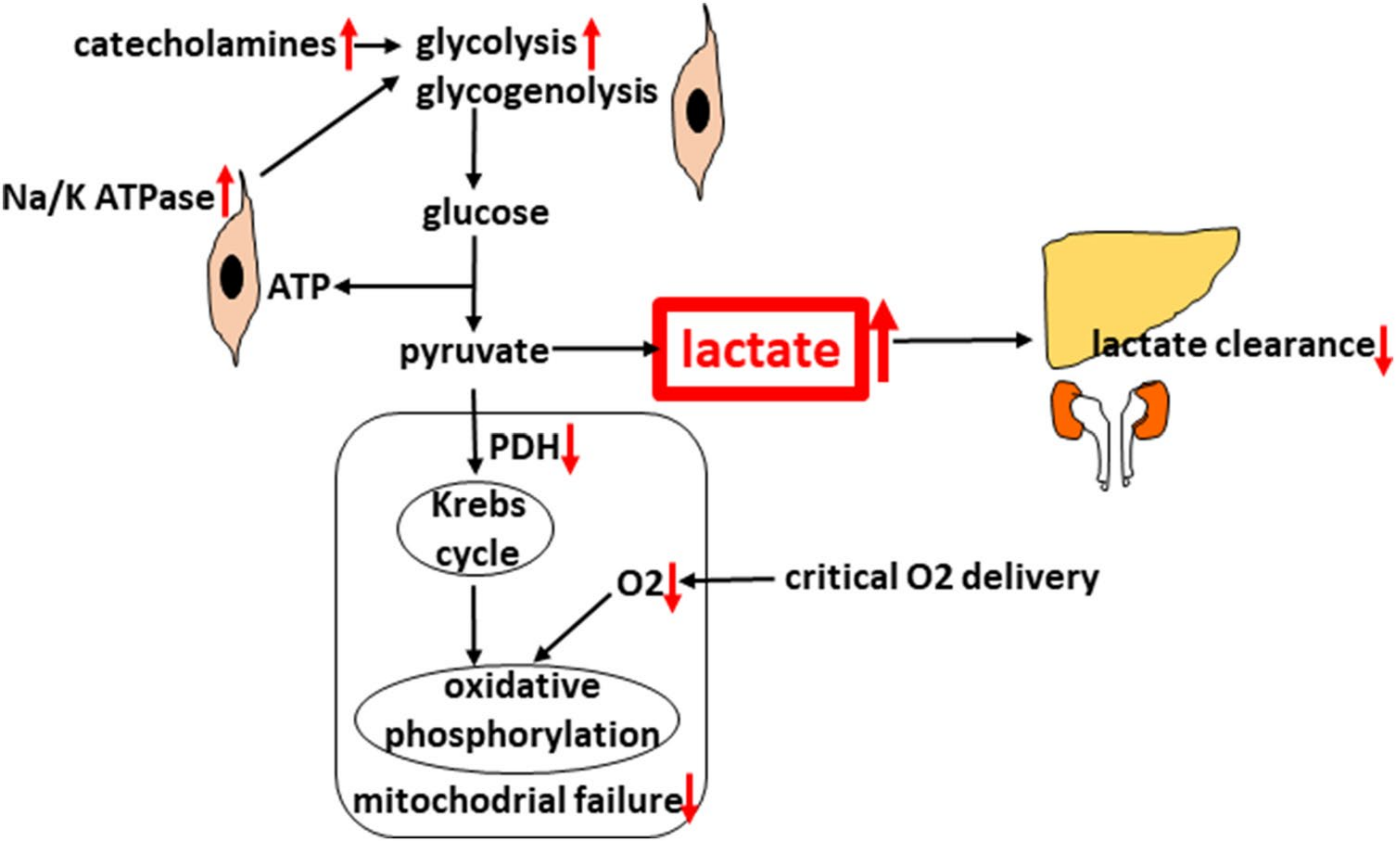
Glucose metabolism and the production of lactate

The glucose molecule is metabolized to pyruvate without the need for O_2_, generating 2 ATP molecules and known as anaerobic glycolysis. In the presence of O_2_, pyruvate enters the mitochondria, where pyruvate dehydrogenase (PDH) converts pyruvate into the acetylkoenzyme A, which enters the Krebs cycle followed by oxidative phosphorylation (1 glucose molecule generates 36 ATP molecules). When available O_2_ drops to critical levels, DO_2_ pyruvate is metabolized by lactate dehydrogenase into lactate. Therefore, lactate is considered to be a marker of anaerobic metabolism. Aside from the hypoxic explanation, however, there are also non-hypoxic pathways for lactate production not related to hypopefusion in shock that are either increased production or decreased clearance. Non-hypoxic production occurs in septic shock due to excessive ß-adrenergic stimulation of muscle cells by intrinsic mechanisms or by the administration of catecholamines [37, 38]. It leads to excessive glycogenolysis and glycolysis. Increased glycolysis produces an abundance of pyruvate that overwhelms the capacity of PDH, thus leading to lactate production. Another reason is malfunction of PDH and other mitochondrial enzymes of aerobic metabolism induced by septic toxins. The liver and kidneys are responsible for clearance of up to 90% of lactate (lactate is converted back to pyruvate by entering the Krebs cycle or is used for gluconeogenesis in the Cori cycle). In case of their hypoperfusion or enzyme failure, clearance is diminished [14, 39] (Fig. 5).

### Measurement

Blood lactate level is routinely measured from blood samples (point-of-care test). Even during the first hours of shock or during decompensation, it is sufficient to measure lactate levels every 1–2 h due to its slower kinetics [6, 28]. There are even systems for continuous invasive monitoring of lactate levels [40].

### Clinical applications

#### Marker of anaerobic metabolism (adequacy of CO)

Lactate informs about perfusion indirectly by reflecting anaerobic metabolism [6]. Because it requires switch of metabolism it is late marker of hypoperfusion not as sensitive in detecting early stages of hypoperfusion as S_v_O_2_, PCO_2_ gap or PCO_2_ gap/C_a–v_O_2_. The cut-off value indicating hypoperfusion is > 2 mmol/l. Lactate exhibits a similar biphasic curvilinear shape of dependence on CO like other parameters, except for septic shock, where the normalization occurs slower (Fig. 2, lactate in septic shock, lactate in non-septic state—curve would be similar to PCO_2_ curve) [41, 42]. First, it is due to non-hypoxic reasons for lactate elevation and, second, to microvascular uncoupling. The correlation between lactate and CO is, therefore, weaker. Improving CO initially causes a rapid drop in lactate, followed by persistent only slowly decreasing lactate levels despite the already normalized perfusion. Therefore, trying to normalize lactate could lead to harmful over-resuscitation by fluid and inotropes [8]. Normalization of PCO_2_ gap and PCO_2_ gap/C_a–v_O_2_ ratio (faster reacting markers of anaerobic metabolism) would suggest that perfusion is normalized and lactate level is elevated for other reasons. Lactate/pyruvate ratio was proposed to discriminate non-hypoxic lactate elevation (> 18 indicates anaerobic metabolism) but it is not widely used due to technical difficulties with measuring pyruvate [14].

#### Prognostic marker and GDT

Lactate is the only parameter to have clear evidence for GDT and is strongly recommended for navigation of treatment by guidelines [6, 28]. Both high value and slow clearance are associated with worse prognosis. Conversely, bringing lactate levels under 2 mmol/l or clearance > 20% every 2 h in the early stage(s) of septic shock (first 8 h) or > 50% in the first 6 h is associated with improved outcomes [1, 6, 43, 44].

#### Marker of distributive shock (uncoupling)

When CO and S_v_O_2_ are normal, increased lactate level can imply microvascular and cellular failure [1, 41].

#### Marker of local hypoxia

Lactate levels can be elevated also if local hypoxia occurs (e.g., acute limb ischemia). Global hypoxia can be ruled out based on other perfusion parameters that would be normal [14].

## PCO_2_ gap (∆PCO_2_, P_v–a_CO_2_)

PCO_2_ gap is the difference between venous and arterial partial pressures of CO_2_.

### Physiological principles

Unlike O_2_, only 5% of CO_2_ is reversibly bound to proteins, mainly HGB (to the amino group creating carbamino HGB). On the other hand, CO_2_ is more physically dissolved in blood than O_2_ because it is 20 times more soluble, but still comprises only 5% of CO_2_ in the blood. The majority (90%) of CO_2_ in blood is in the form of bicarbonate: the CO_2_ originating from tissues combines with water (H_2_O) to form H_2_CO_3_. This takes place mainly in erythrocytes catalyzed by carbonic anhydrase (only a minority of CO_2_ is created slowly uncatalyzed in plasma). H_2_CO_3_ dissociates in erythrocytes into HCO_3_^−^ and H^+^. HCO_3_^−^ leaves the erythrocytes via a bicarbonate/chloride exchanger and is dissolved in blood flowing to the lungs, where the reverse reaction occurs (in erythrocytes and the lung endothelium), catalyzed by carbonic anhydrase and bringing H_2_O and CO_2_ [15]. CO_2_ is highly lipophilic and freely diffuses through membranes and is exhaled by the lungs. The CO_2_ dissociation curve (relation between PCO_2_ and content of CO_2_) is curvilinear (unlike O_2_, which is S-shaped); however, in the physiological range, it is near linear, which is why CO_2_ content can be substituted by PCO_2_ * *k* (dissociation coefficient).

As mentioned above, PCO_2_ gap is the difference between partial pressure of CO_2_ in arterial and venous blood. As described for SvO_2_, the Fick principle can also be applied: CO_2_ production (VCO_2_) is proportional to a flow through the tissues and arteriovenous concentration difference in CO_2_ [45, 46]:

$${\text{VCO}}_{2} = {\text{CO}} \times \left( {{\text{C}}_{{\text{a}}} {\text{CO}}_{2} { } - {\text{ C}}_{{\text{v}}} {\text{CO}}_{2} } \right),$$

where C_a_ and C_v_CO_2_ represent the arterial and venous concentrations of CO_2_, respectively.

Also mentioned above, concentration can by calculated from partial pressure (PCO_2_) as follows:

$${\text{VCO}}_{2} = {\text{CO}} \times k \times \left( {{\text{P}}_{{\text{v}}} {\text{CO}}_{2} - {\text{P}}_{{\text{a}}} {\text{CO}}_{2} } \right) = {\text{CO}} \times k \times {\text{P}}_{{\left( {{\text{v}} - {\text{a}}} \right)}} {\text{CO}}_{2}$$

and

$${\text{PCO}}_{2} \;{\text{gap}} = \frac{{{\text{VCO}}_{2} }}{{{\text{CO}} \times k}}$$

**Fig. 6:**
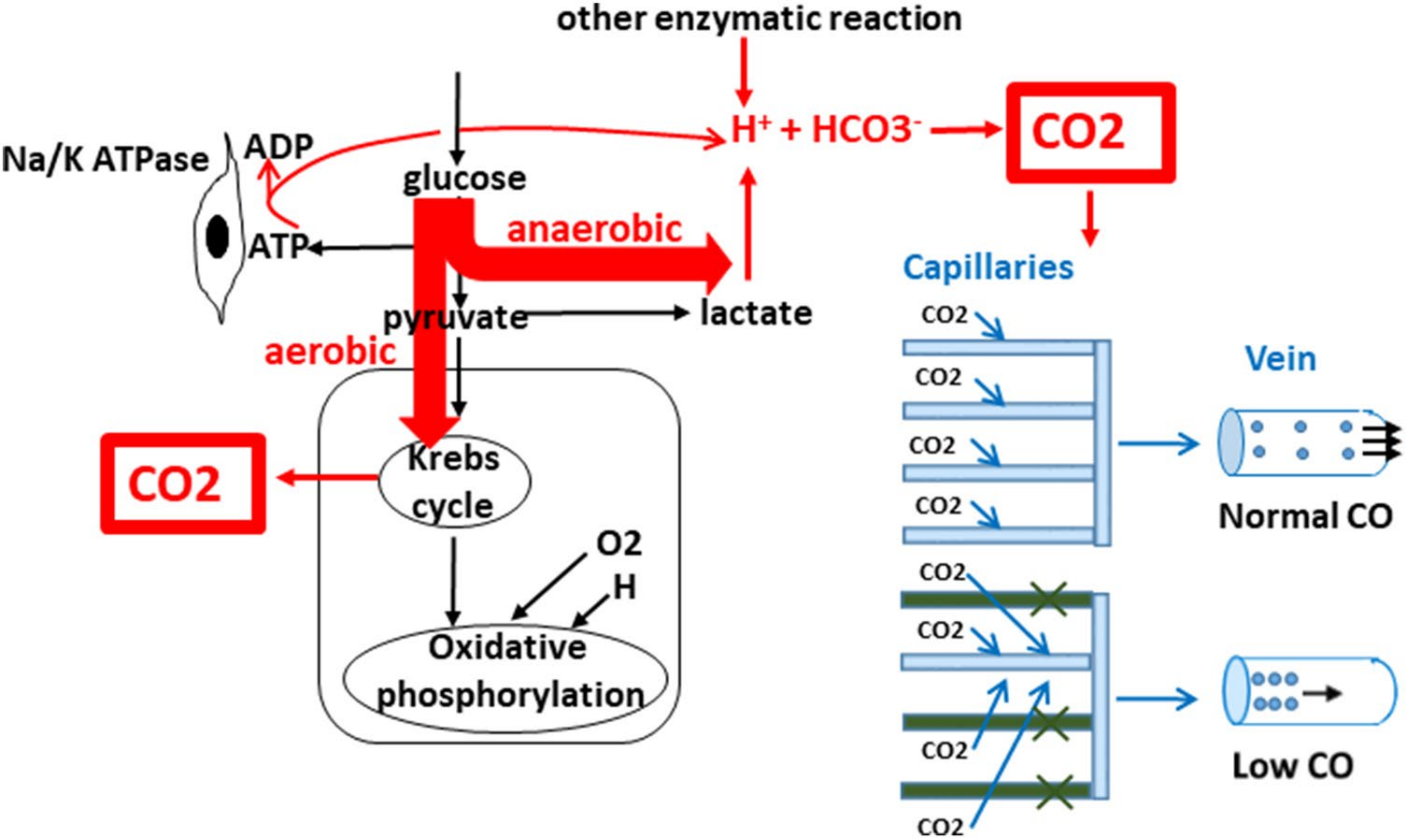
Production of carbon dioxide (CO_2_) and its relationship with cardiac output (CO). All produced CO_2_ easily diffuses and dissolves in blood. Transport from tissues to the venous blood does not restrict clearance from tissues. All produced CO_2_ always gets to the venous blood without accumulation in the tissues, and its concentration in the veins depends only on venous return. Higher CO leads to smaller venous CO_2_ concentration and smaller arteriovenous difference. Lower CO leads to slower flow through capillaries and the entire CO_2_ production is dissolved in smaller venous blood volume, known as the “stagnation phenomenon”. This is why there is higher amount of CO_2_ dissolved in venous blood and higher arteriovenous difference. The same applies to reduced capillary net (despite normal CO) when CO_2_ from areas with damaged net is drained by remaining capillaries leading inevitably to high CO_2_ concentration. HCO_3_^−^, bicarbonate

PCO_2_ gap is proportional to CO_2_ production (VCO_2_) in tissues and inversely related to CO (i.e., flow through the tissues (elimination from tissues) [47]. In normoxemia, aerobic production of CO_2_ occurs in the Krebs cycle. In hypoxemia, VCO_2_ remains relatively stable because, although aerobic production of CO_2_ decreases, it is partly counterbalanced by increased anaerobic production. In fact, VCO_2_ slightly decreases during hypoxia despite anaerobic CO_2_ generation; however, for clinical purposes, it can be considered constant. Anaerobic CO_2_ production comes from increased production of H^+^ buffered by HCO_3_^–^ (further converted to CO_2_). The source of H^+^ is mainly from ATP hydrolysis, then lactate production (although lactate production does not generate H^+^ directly because one H^+^ is generated to make pyruvate from glucose, but one H^+^ is consumed to make lactate from pyruvate), and other enzymes producing H^+^. In normoxemic conditions, H^+^ is consumed in oxidative phosphorylation, which is not the case in anaerobic metabolism [14, 39] (Fig. 6).

This explains why PCO_2_ gap cannot be used to detect hypoxia and anaerobic metabolism—normal PCO_2_ gap does not mean the absence of hypoxia. As mentioned above, because VCO_2_ is essentially constant in normoxia and hypoxia, and because the diffusibility through membranes and solubility in blood is very high (not restricting CO_2_ elimination from tissues), PCO_2_ gap is determined solely by capillary venous outflow (i.e., CO, that clears produced CO_2_ [45]:

$${\text{PCO}}_{2} \;{\text{gap}}\; \uparrow \, = \frac{{{\text{VCO}}_{2} }}{{{\text{CO}}\, \downarrow \, \times \, k}}$$

When DO_2_ was lowered beyond critical value in a canine model, either by reducing blood flow using blood with normal SaO_2_, or by preserved blood flow but with low S_a_O_2_. The former lead to an increase in PCO_2_ gap, whereas the latter did not change PCO_2_ gap [48].

### Difference between P_cv_CO_2_ and P_v_CO_2_

The values of PCO_2_ gap exhibit the same trends as S_v_O_2_ when using mixed venous blood (P_(v–a)_CO_2_) or central venous blood (∆PCO_2_). Again, similar to the case of SvO_2_, the use of central venous blood is widely accepted as a surrogate for calculation of PCO_2_ gap [46, 49].

### Measurement of PCO_2_ gap

PCO_2_ gap is routinely measured from blood samples (point-of-care test); however, there are some limitations. The blood capacity for CO_2_ is increased (dissociation curve is not linear) with low HGB saturation with O_2_ (hypoxia, Haldane effect) and acidosis by carrying more CO_2_ by HGB. In severe hypoxia and acidosis, CO_2_ content can be increased by these factors at a given PCO_2_ and, therefore, calculation of CO_2_ gap from pCO_2_ can be imprecise [14].

### Clinical applications

#### Marker of venous return (adequacy of CO)

Venous content of CO_2_ and PCO_2_ gap depends, in fact, only on microvasculatory venous return (Fig. 6) and PCO_2_ gap reflects venous return from the capillary bed and the adequacy of the microcirculation [14].

In the state of coupling macro- and microcirculation, it indirectly reflects CO and has similar biphasic curvilinear shape of dependence on CO similar to other parameters [50] (Fig. 2). It does not have as robust evidence as other parameters on GDT; however, guidelines have recommended the use of PCO_2_ gap to help assess the adequacy of CO as well as to guide therapy [1]. In normal conditions (normal CO and homogenous healthy capillary bed), all CO_2_ production is rapidly washed out, and venoarterial PCO_2_ gradient is minimal. PCO_2_ gap > 6 mmHg (0.8 kPa) is the cut-off value that implies inadequate CO; in that case, the therapeutic option could be to increase CO with the aim of normalizing PCO_2_ gap. On the other hand, in shock with persistent elevation of lactate levels (see below), normalized PCO_2_ gap will indicate the risk for potentially harmful over-resuscitation using fluid and inotropes. The variation of CO_2_ occurs faster than lactate changes; therefore, it is more sensitive marker to hemodynamic changes [14].

In contrast, in the uncoupling state (distributive shock), there is a weak correlation between PCO_2_ gap and CO (similar to S_v_O_2_) because of decreased functional capillary density, with areas with good and poor perfusion (Fig. 6); this can lead to elevation of venous CO_2_ content and PCO_2_ gap despite normal or high CO. In such situations, some patients may benefit from an increase in CO to supranormal value if signs of hypoperfusion persist [51].

#### Prognostic marker

Persistent elevation of PCO_2_ gap in patients with septic shock has been shown to be associated with worse prognosis [52].

#### Not a marker of hypoxia

As mentioned above, PCO_2_ gap does not indicate the metabolic impact of hypoperfusion—it does not reflect hypoxia [47].

## C_v–a_CO_2_/C_a–v_O_2_ ratio

### Physiological principles

This ratio is derived from the RQ, which reflects the ratio of moles of CO_2_ generated per mole of O_2_; it can be directly measured by calorimetry and expressed by the equation:

$${\text{RQ}} = \frac{{{\text{VCO}}_{2} }}{{{\text{VO}}_{2} }}$$

However, it can be also calculated based on the Fick principle:

$${\text{RQ}} = \frac{{{\text{CO}} \times \left( {{\text{C}}_{{\text{v}}} {\text{CO}}_{2} - {\text{C}}_{{\text{a}}} {\text{CO}}_{2} } \right)}}{{{\text{CO}} \times \left( {{\text{C}}_{{\text{a}}} {\text{O}}_{2} - {\text{C}}_{{\text{v}}} {\text{O}}_{2} } \right)}}{ } = { }\frac{{{\text{C}}_{{{\text{v}} - {\text{a}}}} {\text{CO}}_{2} }}{{{\text{C}}_{{{\text{a}} - {\text{v}}}} {\text{O}}_{2} }}$$

Using the partial pressure of CO_2_, it can be obtained a surrogate of RQ:

$${\text{RQ}} = \frac{{{\text{P}}_{{{\text{v}} - {\text{a}}}} {\text{CO}}_{2} }}{{{\text{C}}_{{{\text{a}} - {\text{v}}}} {\text{O}}_{2} }} = \frac{{{\text{PCO}}_{2} \;{\text{gap}}}}{{{\text{C}}_{{{\text{a}} - {\text{v}}}} {\text{O}}_{2} }}$$

In aerobic metabolism, one O_2_ molecule leads approximately to the production of one CO_2_ molecule, and RQ = 1. In hypoperfusion leading to anaerobic metabolism, there is a decline in both VO_2_ and VCO_2_; however, this decline is asymmetric. As mentioned above, VCO_2_ decreases only slightly due to counterbalancing of the aerobic production decrease by an increase in anaerobic production:

$${\text{RQ}}\; \uparrow \, = \frac{{{\text{VCO}}_{2} \; \downarrow }}{{{\text{VO}}_{2} \; \downarrow \downarrow \downarrow }}$$

**Fig. 7:**
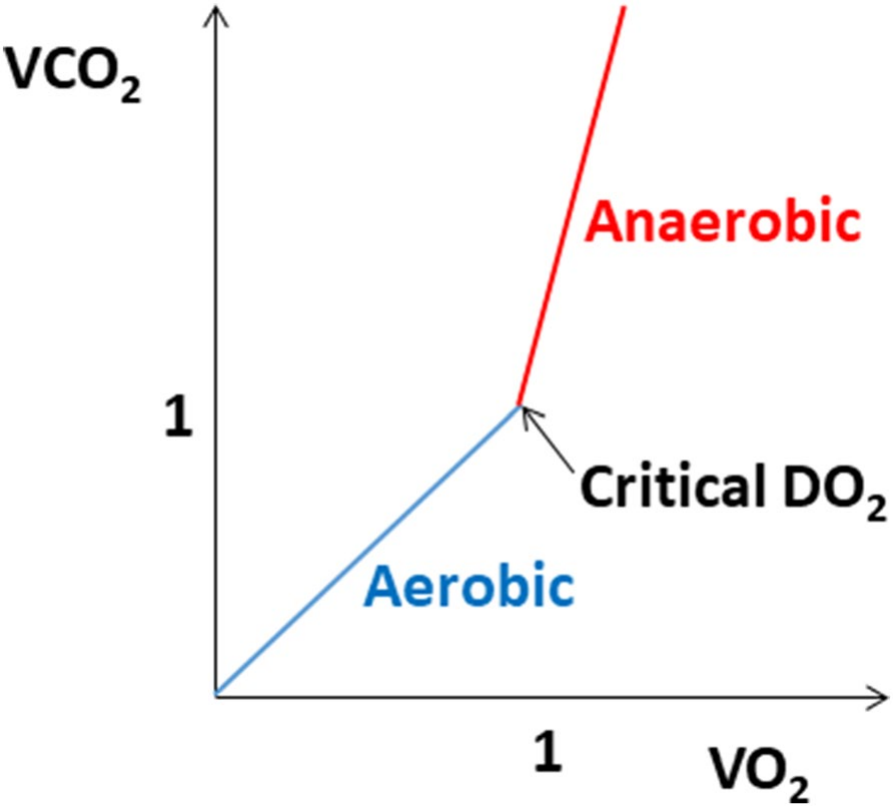
Relationship between carbon dioxide output (VCO_2_) and oxygen consumption (VO_2_) (the respiratory quotient) under aerobic and anaerobic metabolism. DO_2_, oxygen delivery

Therefore RQ > 1 implies a switch to anaerobic metabolism (Fig. 7).

The C_v–a_CO_2_/C_a–v_O_2_ ratio appears to correspond with lactatemia and RQ measured by calorimetry; however, according to some trials, the surrogate PCO_2_ gap/C_a–v_O_2_ ratio may be imprecise due to the Haldane effect [14, 45].

### Clinical applications

#### Marker of anaerobic metabolism (adequacy of CO)

A PCO_2_ gap/C_a–v_O_2_ > 1.4 implies anaerobic metabolism. Its advantage compared to lactate is an earlier reaction [45]. The elevation of both PCO_2_ gap/C_a–v_O_2_ and lactate strongly indicate ongoing anaerobic metabolism. If PCO_2_ gap/C_a–v_O_2_ is elevated but lactate levels are normal, it can suggest the onset of anaerobic metabolism (early stage[s] of shock). If PCO_2_ gap/C_a–v_O_2_ is normal but elevated lactate levels persist, it suggests resolution of aerobic metabolism with persistent lactate elevation from non-hypoxic causes (see above), and over-resuscitation with fluids and inotropes is discouraged [14, 53].

#### Prognostic marker

It has been shown that patients with septic shock and increased PCO_2_ gap/C_a–v_O_2_ have a worse prognosis [54]. In contrast to lactate levels, evidence supporting PCO_2_ gap/C_a–v_O_2_ for GDT is lacking.

#### Marker of distributive shock (uncoupling)

When CO and S_v_O_2_ are high, increased lactate and PCO_2_ gap/C_a–v_O_2_ can imply microvascular and cellular failure [55].

## Algorithm for assessment and monitoring of microcirculation and tissue perfusion

**Fig. 8:**
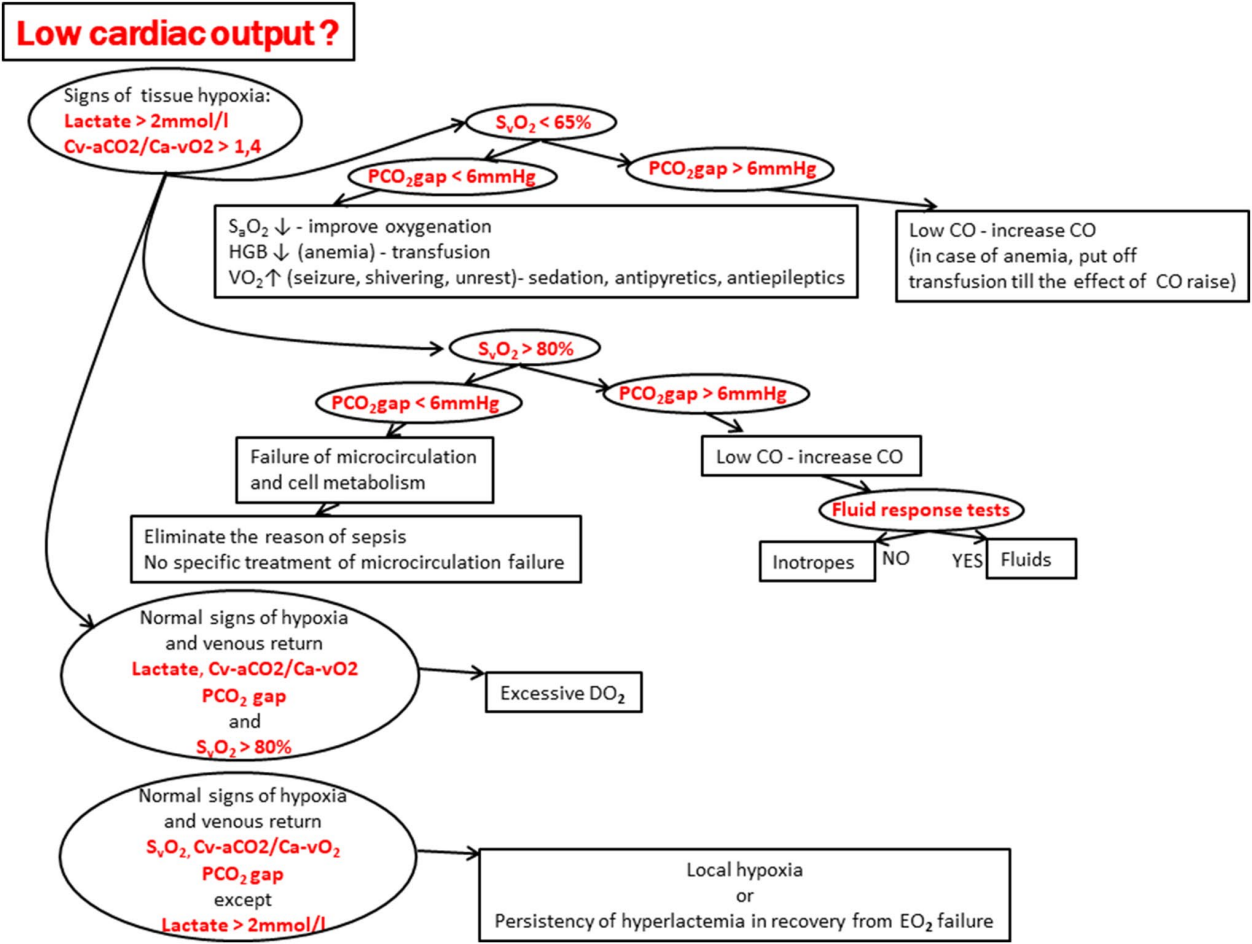
Algorithm for the use of parameters of global oxygen metabolism ( adapted from Mallat et al. [45] and Vallet et al. [56]). C_a–v_O_2_, central venous-to-arterial oxygen difference; CO, cardiac output; DO_2_, oxygen delivery; HGB, hemoglobin; PCO_2_ gap, central venous–arterial carbon dioxide difference; S_a_O_2_, oxygen saturation; S_v_O_2_, mixed venous oxygen saturation; VO_2_, oxygen consumption; ↑, increase; ↓, decrease

The abovementioned biochemical parameters of global O_2_ metabolism are used as clinical markers of different aspects of the microcirculation and tissue perfusion status (Table 2). The precise analysis and accurate interpretation of the measured values enable the recognition of the specific cause of tissue hypoperfusion and optimize the therapeutic intervention. We propose an algorithm for the evaluation of tissue perfusion and microcirculation status and related therapeutic consequences that are based on the findings (Fig. 8).

**Table 2:** Summary of the clinical use of selected biochemical parameters of global oxygen (O_2_) metabolism for the assessment of microcirculation and tissue perfusion

Parameter	Clinical application as a marker	Cut-off value(s)
S_v_O_2_	Hypoxia Extraction O_2_ from hemoglobin Microcirculation and cell failure (high S_v_O_2_)	< 65% > 80%
Lactate	Hypoxia Anaerobic metabolism Strongest data for GDT Also detects local hypoxia	> 2 mmol/l
PCO_2_ gap	Venous return—perfusion	> 0.8 kPa (> 6 mmHg)
PCO_2_ gap/C_a–v_O_2_	Hypoxia Anaerobic metabolism	> 1.4

C_a–v_O_2_, central venous-to-arterial CO_2_ difference; GDT, goal-directed therapy; PCO_2_ gap, central venous–arterial carbon dioxide difference; S_v_O_2_, mixed venous oxygen saturation

## Conclusion

The assessment and monitoring of the microcirculation and tissue perfusion is extremely important in conditions involving circulatory shock. Whereas parameters of the macrocirculation, such as blood pressure or CO, are relatively easily available and are amenable to simple interpretation, the situation at the microcirculatory level is significantly more complex. Moreover, there is often only very limited correlation between the findings at the macrocirculation and microcirculation levels, and therapies directed at simply normalizing the macrocirculation without the knowledge of the status of the microcirculation can be even harmful. Currently, the available methods for evaluating the microcirculation are very limited. In recent years, biochemical markers of global O_2_ metabolism have become routinely used in the assessment of tissue perfusion. However, the interpretation of these values must be based on knowledge of physiological principles and in the context of other findings. Nevertheless, there is mounting evidence that accurate assessment and monitoring of tissue perfusion using parameters of global O_2_ metabolism is essential for the selection of the most appropriate therapeutic strategy and may improve therapeutic outcomes in patients with critical circulatory conditions.

## Data Availability

Not applicable.

## Abbreviations

∆PCO_2_: Venous–arterial carbon dioxide difference from central venous blood
C_a_O_2_: Arterial oxygen concentration
C_a–v_O_2_: Arterial–venous difference in oxygen concentration
CO: Cardiac output
CVC: Central venous catheter
C_v_O_2_: Venous oxygen concentration
DO_2_: Oxygen delivery
EO_2_: Oxygen extraction
GDT: Goal-directed therapy
HGB: Hemoglobin
HR: Heart rate
NIRS: Near-infrared spectroscopy
PAC: Pulmonary artery catheter
P_a_CO_2_: Partial pressure of arterial carbon dioxide
P_a_O_2_: Partial pressure of arterial oxygen
PCO_2_ gap: Venous–arterial carbon dioxide difference
P_cv_CO_2_: Partial pressure of central venous carbon dioxide
PDH: Pyruvate dehydrogenase
P_v–a_CO_2_: Venous–arterial carbon dioxide partial pressure difference
P_v_CO_2_: Partial pressure of mixed venous carbon dioxide
RQ: Respiratory quotient
rSO_2_: Peripheral oxygen saturation
S_a_O_2_: Arterial oxygen saturation
S_cv_O_2_: Central venous oxygen saturation
SV: Stroke volume
S_v_O_2_: Mixed venous oxygen saturation
VCO_2_: Carbon dioxide production
VO_2_: Oxygen consumption
